# Social and Structural Determinants of Lower Extremity Amputations in Diabetes

**DOI:** 10.1007/s11892-025-01598-y

**Published:** 2025-07-02

**Authors:** Misty D. Humphries

**Affiliations:** Division of Vascular Surgery, UC Davis Health Heart and Vascular Center, UC Davis Advanced Wound Care Program, Vascular Residency, UC Davis Health, 2335 Stockton Blvd, NAOB 5017, Sacramento, CA 95811 USA

**Keywords:** Limb ischemia, Leg amputation, Access, Disparities

## Abstract

**Purpose of Review:**

Lower extremity amputations (LEAs) are among the most severe complications of diabetes, with approximately 1.5 million procedures performed globally each year. This review explores the impact of social and structural determinants of health on amputation rates in diabetic patients, highlighting disparities driven by systemic factors.

**Recent Findings:**

Structural determinants such as healthcare policies and economic systems intersect with social factors, including access to care, racial disparities, and socioeconomic status, influencing amputation risk. Black patients with diabetes face up to a fourfold increased risk of major amputation compared to non-Hispanic white patients. Lower socioeconomic status is also strongly linked to higher amputation rates. Geographic and environmental factors, like food deserts and limited access to specialized care, further exacerbate these disparities. Emerging prevention strategies, such as telemedicine and mobile health units, demonstrate promise in improving access to care.

**Summary:**

Addressing disparities in LEAs requires comprehensive policy changes and targeted interventions. Future directions include leveraging artificial intelligence and precision medicine alongside community-based programs to reduce amputation rates in high-risk diabetic populations.

## Introduction

Lower extremity amputations are among the most devastating complications of diabetes, linked to high rates of disability, worse quality of life, and increasing mortality, not to mention severe economic burden. Advancements in vascular surgery and medical management have improved outcomes for diabetic patients with peripheral artery disease (PAD), coronary artery disease (CAD), and cerebrovascular disease (CVD); however, the burden of LEAs disproportionately affects vulnerable populations. Understanding the role of social and structural determinants is crucial for developing effective, equitable strategies to prevent LEAs in diabetic patients.

### Epidemiology of Diabetes and Lower Extremity Amputations

Approximately 1.5 million LEAs are performed annually worldwide, with diabetes accounting for 50–70% of these cases [1]. The global health burden of PAD is increasing with substantial variations in incidence across different populations and geographic regions [2]. In the United States, the age-adjusted incidence of LEAs in diabetic individuals declined by 43% between 2000 and 2010 [3]. Work that has looked at amputation trends for high-risk patients with wounds, however, has shown that those with combined diabetes and peripheral artery disease have the highest risk of amputation, with rates that are increasing over time [4]. 

### Structural Versus Social Determinants of Health

Structural determinants of health, encompassing overarching systems like governance, economic policies, and societal norms, serve as the fundamental drivers of social determinants.^1^ These macro-level forces shape the distribution of resources, power, and opportunities within a society, ultimately determining the social conditions in which individuals live, work, and age [5]. For instance, policies that perpetuate economic inequality, such as regressive taxation or limited access to quality education, create the conditions for social determinants like poverty, unemployment, and low educational attainment.^2^ These social determinants, in turn, directly impact health outcomes through pathways such as reduced access to healthcare, healthy food, and safe housing. Therefore, understanding the influence of structural determinants is crucial for comprehending the root causes of health inequities and developing effective interventions.

Social determinants of health, such as access to quality education, safe housing, and nutritious food, act as mediators between structural determinants and health outcomes.^4^ While structural determinants establish the overall social and economic context, social determinants represent the specific pathways through which these structural forces impact individual and population health [6]. For example, discriminatory housing policies (structural determinant) can lead to residential segregation and concentrated poverty (social determinants), which in turn increase exposure to environmental hazards, violence, and chronic stress, ultimately contributing to poorer health outcomes. Therefore, addressing social determinants is essential for mitigating the adverse health effects of structural inequities.

**Fig. 1:**
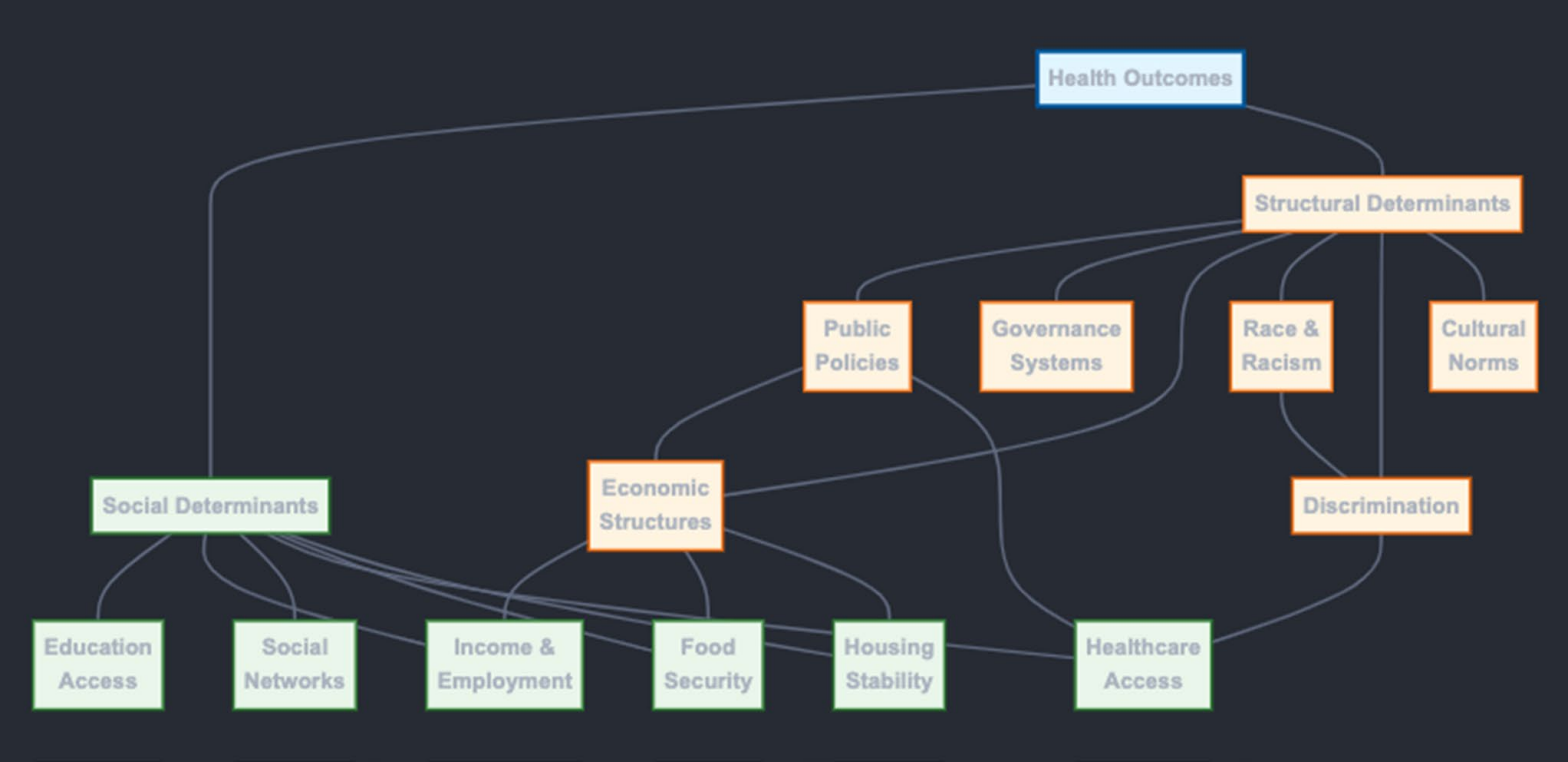
The connection between structural and social determinants of health

Social and structural determinants of health are not independent entities but rather exist in a dynamic and interdependent relationship.^5^ Structural determinants shape the social conditions in which individuals live. In contrast, social determinants, in turn, can influence the evolution of structural determinants through social movements, political mobilization, and community organizing [7]. For instance, persistent health inequities stemming from social determinants like poverty and lack of access to healthcare can galvanize social movements demanding policy changes that address the underlying structural determinants, such as advocating for living wages or universal healthcare coverage. This interplay highlights the need for comprehensive approaches that simultaneously address social and structural determinants to achieve health equity (Fig. 1).

### Racial and Ethnic Disparities

Race is a social construct with no biological basis. The experience of race, shaped by historical and ongoing racism, and the experience of ethnicity, influenced by cultural and societal factors, both operate as social and structural determinants of health. Significant racial disparities persist in major amputation rates among diabetes patients in the United States, with Black patients facing disproportionately higher risks. Black patients with diabetes are up to four times more likely to undergo major amputation compared to non-Hispanic white patients, even when controlling for demographic factors like age and gender [8]. These disparities extend to diabetes management itself, with Hispanic/Latino individuals showing 46% higher odds of poor glycemic control (OR: 1.46; 95% CI, 1.16–1.83) and Black individuals showing 28% higher odds (OR: 1.28; 95% CI, 1.04–1.57) compared to non-Hispanic whites.

Inequitable access to preventive care and specialty services drives these disparate outcomes in amputation rates. Research by Taylor et al. found that high-risk Black and Hispanic patients with diabetic foot ulcers received fewer visits with specialists and were less likely to receive timely specialist care compared to white patients. The same study demonstrated that earlier specialist referral significantly lowered amputation rates, highlighting how delayed access to preventive services directly contributes to these inequities [9]. Furthermore, Black and Hispanic patients face additional barriers to limb preservation, as they are less likely to undergo revascularization procedures that could prevent amputation [10]. When researchers factored in social determinants, particularly food security, these disparities slightly decreased, with Hispanic/Latino and Black populations showing odds ratios of 1.39 (95% CI, 1.08–1.81). Interestingly, when healthcare access and behavioral health factors were considered, the disparities increased, particularly for Hispanic/Latino individuals (OR: 1.63; 95% CI, 1.24–2.16). The disparities remained substantial even among those with private insurance coverage (OR: 1.66; 95% CI, 1.10–2.52) [11]. 

### Access to Quality Healthcare

Diabetic foot complications, including ulcers and amputations, are not merely consequences of the disease itself but rather reflect deep-seated inequities in access to healthcare. Structural determinants, such as the unequal distribution of healthcare providers and limited insurance coverage, create significant barriers to timely and effective diabetes management. Many individuals, particularly those in low-income communities and rural areas, face a scarcity of specialized providers like podiatrists and endocrinologists, limiting their access to essential preventative care and early intervention for foot complications [12]. This is further compounded by inadequate insurance coverage, particularly for those reliant on Medicaid, which may have restrictive provider networks or insufficient reimbursement policies for preventative foot care and advanced wound management therapies. These structural barriers create a system where individuals with limited resources are disproportionately affected by preventable complications, perpetuating a cycle of health disparities.

These structural barriers translate into profound challenges for individuals managing diabetes. Lack of regular foot exams and timely detection of neuropathy or vascular issues, often due to limited access to specialists or financial constraints, can lead to delayed treatment of diabetic foot ulcers. As highlighted in studies focused on early diagnosis, inconsistent access to specialized care hinders proper ulcer management and infection control, increasing the risk of amputation [13]. Furthermore, inadequate insurance coverage and high medication costs can hinder adherence to essential medications for diabetes management, further contributing to the development and progression of foot complications [14]. Addressing these intertwined structural and social determinants requires a multi-faceted approach that includes policy interventions to expand insurance coverage, incentivize provider participation in underserved areas, and community-based programs that address social determinants and empower patients to manage their diabetes effectively.

### Socioeconomic Status

 Socioeconomic status (SES) measures an individual’s or family’s combined economic and social position in society relative to others. It is shaped by the policies that result in income inequality and limit educational opportunities. The most crucial structural influencer of socioeconomic status is employment, which is much more than just the exchange of work for compensation today. Patients with diabetes have more than twice the number of absentee days per year compared to those without. Patients with complicated diabetes, such as those with neuropathy, lose as much as 26 days of productivity per year [ 15 ]. This results in decreased income; for every $10,000 decrease in median household income, amputation rates increase by 4.4% (95% CI, 3.9–4.8%).

Lower SES is frequently associated with lower health literacy. In a 2024 study of health literacy representing 10 million Americans with diabetes, 63% had poor health literacy [16]. Since health literacy is a strong predictor of using information to make treatment choices, including when to seek medical care, it is no surprise that diabetic patients who require lower extremity amputation are 8 times more likely to have low health literacy [17]. Socioeconomic status also plays a crucial role in shaping individuals’ ability to engage in self-care practices. While the word self-care can be interpreted in many ways, in the context of chronic diseases such as diabetes, it also refers to healthy eating, routine physical activity, medication adherence, stress mitigation, and diligent foot care. Many patients who require amputation due to diabetes don’t have the time or capacity for this type for this type of self-care [18]. 

### Geographic and Environmental Factors

Geographic and environmental factors significantly influence a patient’s ability to manage diabetes by shaping daily behaviors, stress levels, and access to essential resources. In communities showing more significant economic distress, as measured by a 10-point increase in the Distressed Communities Index, which characterizes access to healthy food, inadequate public transportation, and fewer healthcare facilities, amputation rates have been shown to rise by 3–16% [16]. Living in areas with limited availability of healthy foods, such as food deserts, can force reliance on processed, high-calorie options that worsen glycemic control and double the risk of wound complications after surgery, with the most common complication being infection [19, 20]. Infection, in turn, increases the risk of amputation [21]. Similarly, neighborhoods lacking safe spaces for physical activity, like parks or well-maintained sidewalks, hinder the ability to exercise, a cornerstone of diabetes management. Environmental stressors, such as exposure to crime, pollution, or unstable housing, may exacerbate stress-related hormone fluctuations that impair glucose regulation [22]. One factor garnering more attention is extreme climates or natural disasters. Emergency conditions can disrupt routine diabetes management by making it challenging to store insulin safely or access stable electricity for medical devices. These can also disrupt the production of drugs for diabetes, leading to public health crises for millions of patients. These geographic and environmental determinants can often compound and create significant barriers for patients to keep their Hbg A1c under control.

### Prevention and Management

Current evidence supports targeted screening and prevention programs in high-risk communities. Successful interventions have incorporated telemedicine and mobile health units to reach underserved populations with diabetes to decrease amputation outcomes. Asynchronous telemedicine facilitates remote glucose monitoring on digital platforms such as mobile apps, allowing patients to engage continuously and improving adherence to lifestyle modifications and medication regimens [23]. These platforms often integrate with wearable devices, providing real-time data to both patients and clinicians. Synchronous telemedicine, which may be more resource-intensive, uses virtual consultations and allows patients in rural areas to receive specialty care unavailable in their community. This enables timely treatment plan adjustments or expedition of care without in-person visits and can decrease the risk of lower extremity amputation [24]. This is particularly valuable for patients in rural areas or those with mobility/transportation challenges.

Mobile health units (MHUs) and targeted screening programs improve care. Both increase access to essential diabetes screenings, such as HbA1c tests and retinal exams, by traveling to areas with limited healthcare infrastructure or setting up a pop-up clinic in areas devoid of specialists, such as vascular deserts. These modalities can provide on-site education and counseling on nutrition, physical activity, and medication adherence tailored to the community’s needs [25]. These educational services empower patients to manage their condition effectively. Finally, MHUs and target screening programs foster community trust by employing local healthcare workers, offering culturally competent care, and addressing language and cultural barriers [26]. 

Guideline-directed medical therapy (GDMT), access to newer medications, and revascularization procedures to prevent limb loss have all been studied in diabetic patients, and data shows that there are evident disparities. Racial/ethnic minority groups and those with lower socioeconomic status are less likely to receive GDMT for peripheral artery disease [27]. The use of sodium-glucose cotransporter 2 (SGLT2) inhibitors and glucagon-like-peptide-1 receptor agonists, which has been associated with reduced cardiovascular and major adverse limb-related events (MALE), is also lower in diabetic patients with disparities of care. There are marked disparities in access to both endovascular and surgical revascularization options, with studies demonstrating lower rates of limb salvage procedures and higher rates of major amputation among underserved populations [28]. These disparities are often exacerbated by inadequate health insurance coverage, limited access to specialized vascular care centers, delayed presentation due to barriers in primary care access, and implicit bias within healthcare systems. The impact of these treatment gaps is particularly concerning, given that diabetes patients already face an elevated baseline risk of limb loss, cardiovascular events, and mortality, making timely and appropriate intervention crucial for optimizing outcomes.

### Future Directions and Research Priorities

Here’s a revised Future Directions section that reduces redundancy and better focuses on innovative solutions:

### Future Directions and Research Priorities

Current literature emphasizes the need for innovative interventions that address both social and structural determinants of diabetic complications. Priority areas include community-based interventions, precision medicine, digital health technologies, and large-scale policy changes. While community-based initiatives like culturally tailored education programs and mobile screening clinics show promise, significant work remains to optimize their implementation in underserved communities. Programs combining traditional medical care with social support services through community health workers demonstrate potential but require rigorous evaluation to maximize impact. Critical research gaps persist in understanding how to scale these programs effectively while addressing cultural barriers and enhancing healthcare navigation across diverse populations.

Integrating artificial intelligence and wearable technology offers transformative possibilities for reducing amputation risk, particularly among vulnerable populations. Advanced algorithms can analyze data from continuous glucose monitors and other wearable devices to predict complications before they occur, enabling early intervention. These technologies can overcome traditional barriers by providing personalized health management tools in multiple languages, with user-friendly interfaces that account for varying levels of health literacy. For instance, AI-powered systems can automatically adjust insulin dosing recommendations based on individual patterns while simultaneously alerting healthcare providers to trends that may indicate increased amputation risk.

The future of amputation prevention also lies in precision medicine approaches that consider biological and social risk factors. Research is needed to understand how genetic factors interact with social determinants to influence amputation risk, potentially leading to more targeted interventions. Investigating novel payment models that incentivize prevention and comprehensive care coordination could help healthcare systems better serve at-risk populations. These research priorities, combined with policy initiatives that address underlying structural barriers, represent the most promising path toward reducing the burden of diabetes-related amputations across all communities.

## Conclusion

The prevention of diabetes-related lower extremity amputations requires a fundamental shift in how we approach both healthcare delivery and social equity. Evidence demonstrates that amputation rates reflect broader societal inequities, with vulnerable populations bearing a disproportionate burden of this devastating complication. While technological advances and improved medical treatments offer promise, a meaningful reduction in amputation rates will require addressing underlying structural and social determinants of health. Success demands a multi-faceted approach that combines policy reform, enhanced healthcare access, and innovative care delivery models with particular attention to underserved communities. Future research must focus on implementing and scaling interventions that address medical and social factors while ensuring equitable access to preventive care. Only through comprehensive efforts that tackle both the medical and social aspects of diabetes care can we hope to reduce the burden of amputations across all populations.This article does not contain any studies with human or animal subjects performed by any of the authors.

## Key Reference

* Describes the diabetes problem currently and in the future.

Sun H, Saeedi P, Karuranga S, Pinkepank M, Ogurtsova K, Duncan BB, et al. IDF Diabetes Atlas: Global, regional and country-level diabetes prevalence estimates for 2021 and projections for 2045. Diabetes Res Clin Pract. 2022 Jan 1;183:109119.

* Explains racial and ethnic disparities in diabetes treatment.

Zakaria NI, Tehranifar P, Laferrère B, Albrecht SS. Racial and Ethnic Disparities in Glycemic Control Among Insured US Adults. JAMA Netw Open. 2023 Oct 5;6 (10):e2336307.

* Addresses location and living area in health disparities.

Mujahid MS, Maddali SR, Gao X, Oo KH, Benjamin LA, Lewis TT. The Impact of Neighborhoods on Diabetes Risk and Outcomes: Centering Health Equity. Diabetes Care. 2023 Sep;46 (9):1609–18.

* Demonstrates how various areas have different disparities.

DiLosa K, Humphries MD, Molina VM, Daniele T, Tiu MD, O’Banion LA. Using Vascular Deserts as a Guide for Limb Preservation Outreach Programs Successfully Targets Underserved Populations. Ann Vasc Surg. 2024 Dec 1;109:238–44.

## Data Availability

No datasets were generated or analysed during the current study.
